# Unhealthy herds and the predator–spreader: Understanding when predation increases disease incidence and prevalence

**DOI:** 10.1002/ece3.9918

**Published:** 2023-03-24

**Authors:** Robert L. Richards, Bret D. Elderd, Meghan A. Duffy

**Affiliations:** ^1^ Department of Biological Sciences Louisiana State University Baton Rouge Louisiana USA; ^2^ Department of Ecology & Evolutionary Biology University of Michigan Ann Arbor Michigan USA

**Keywords:** healthy herds, parasitoid, pathogen, predator–host–parasite interactions, predator–prey–parasite interactions, predator–spreader, tritrophic interaction

## Abstract

Disease ecologists now recognize the limitation behind examining host–parasite interactions in isolation: community members—especially predators—dramatically affect host–parasite dynamics. Although the initial paradigm was that predation should reduce disease in prey populations (“healthy herds hypothesis”), researchers have realized that predators sometimes increase disease in their prey. These “predator–spreaders” are now recognized as critical to disease dynamics, but empirical research on the topic remains fragmented. In a narrow sense, a “predator–spreader” would be defined as a predator that mechanically spreads parasites via feeding. However, predators affect their prey and, subsequently, disease transmission in many other ways such as altering prey population structure, behavior, and physiology. We review the existing evidence for these mechanisms and provide heuristics that incorporate features of the host, predator, parasite, and environment to understand whether or not a predator is likely to be a predator–spreader. We also provide guidance for targeted study of each mechanism and quantifying the effects of predators on parasitism in a way that yields more general insights into the factors that promote predator spreading. We aim to offer a better understanding of this important and underappreciated interaction and a path toward being able to predict how changes in predation will influence parasite dynamics.

## Graphical abstract

Although the initial paradigm was that predation should reduce disease in prey populations (“healthy herds hypothesis”), researchers have realized that predators sometimes increase disease in their prey. These “predator‐spreaders” are now recognized as critical to disease dynamics, but empirical research on the topic remains fragmented. We review the existing evidence for these mechanisms and provide heuristics that incorporate features of the host, predator, parasite, and environment to understand whether or not a predator is likely to be a predator‐spreader.

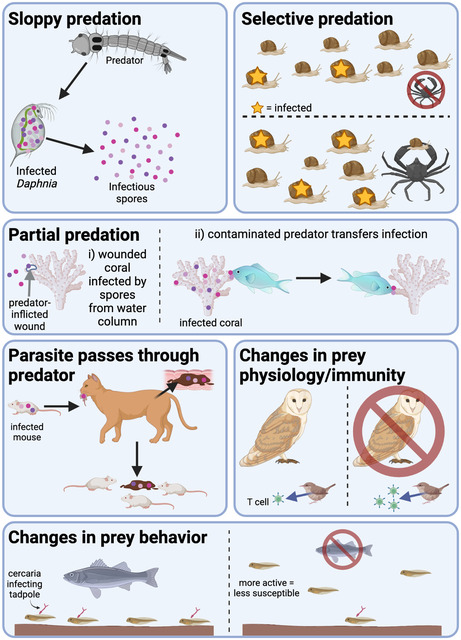

## 1 INTRODUCTION

Because food web members can dramatically impact host–parasite dynamics through a wide variety of mechanisms, disease ecologists now recognize the limitation of examining host–parasite interactions in isolation. This potential for food web members to alter host–parasite dynamics is central to the healthy herds hypothesis, which posits that predators can substantially decrease parasitism in their prey by directly consuming infected individuals (Packer et al., 2003). The formalization of this hypothesis spurred decades of subsequent empirical work testing this prediction with a variety of systems and study designs. Perhaps surprisingly, the net outcome of this work has been the realization that the effect of predators on parasites in their prey is highly variable (Duffy et al., 2019; Lopez & Duffy, 2021; Richards et al., 2022). In fact, predators often *increase* parasitism in their prey, raising the question: Is it possible to predict a priori what the impact of predation will be for a particular predator–prey–parasite system? Being able to make these predictions is both of fundamental ecological interest and potential conservation importance.

Predation and parasitism are known to interact in a variety of ways. Predators control parasites by eating susceptible or infected hosts, and make it harder for parasites to persist by reducing host density. Moreover, predation on free‐living parasite stages can have strongly negative effects on disease (Johnson et al., 2010). However, predators can also be key to the transmission of biologically important parasites. Trophically transmitted parasites require predation of infected intermediate hosts by definitive hosts in order to complete their life cycle, resulting in parasites increasing with predator presence (Kuris, 2003; Lafferty, 1999). Similarly, vector‐transmitted parasites, including *Plasmodium falciparum* which causes malaria in humans, depend on partial‐ or micropredation by insect vectors in order to infect those vectors, complete their life cycle, and infect more vertebrate hosts. In both of these latter examples of predators increasing parasitism the predator itself is a host of the parasite, further complicating these interactions. The role of predators in vector and trophic transmission is well understood. In systems where predators do not serve as hosts, prior work has begun to develop a taxonomy of mechanisms by which these non‐host predators can impact parasitism in their prey (Duffy et al., 2019; Lopez & Duffy, 2021). However, the field lacks a clear framework for understanding the mechanisms by which non‐host predators often increase parasitism in contrast to the healthy herds hypothesis, a phenomenon that appears common and potentially of broad relevance to wildlife and human health. This lack of an understanding of when predators should be expected to make prey sicker is a problem because a recent meta‐analysis found that one of the most important factors determining whether predators increase disease in their prey is whether the predator was identified by researchers as being a “predator–spreader” (Richards et al., 2022); this raises the question of whether it is possible to predict a priori if something is likely to be a predator–spreader.

Although the depth of our understanding of the mechanisms by which non‐host predators increase parasitism in their prey varies, it is clear that a range of mechanisms can produce this outcome, even in a single system (e.g., in zooplankton (Duffy et al., 2019)). The variety of studies of predators increasing parasitism in their prey pales in comparison to the number of unique mechanisms by which predators may positively influence parasitism (Buss & Hua, 2018; Cáceres et al., 2009; Duffy et al., 2019; Holt & Roy, 2007; Lopez & Duffy, 2021; Richards et al., 2022; Stephenson et al., 2015), making an assessment of the most common mechanisms and the circumstances under which they occur difficult. However, these mechanisms of predator spreading exist along a spectrum that we can use to help us understand when predators might increase parasitism in their prey.

**FIGURE 1:**
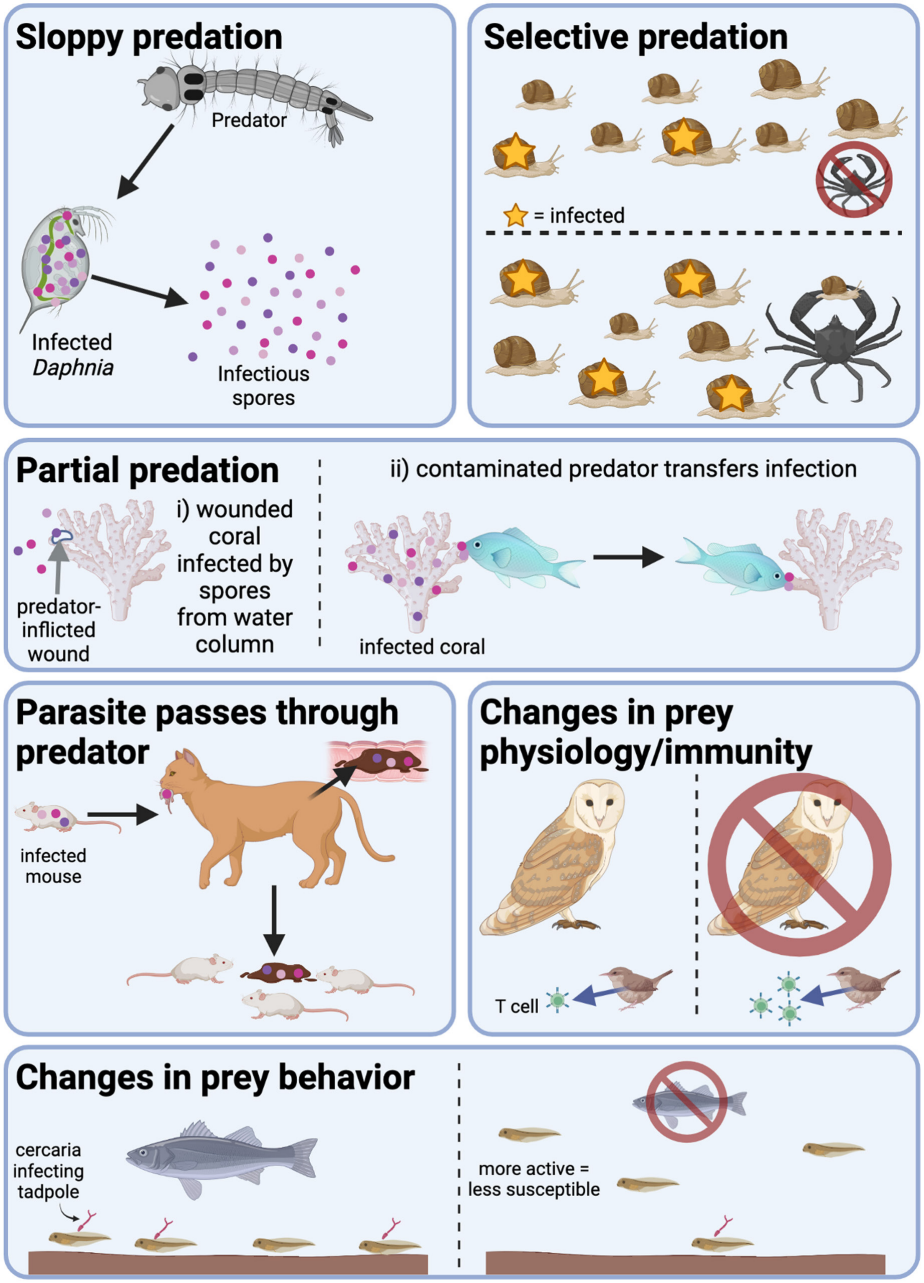
Six mechanisms of predator spreading. The main mechanisms by which predators facilitate parasite transmission and infection, discussed in detail in the main text, and depicted here. Created with BioRender.com.

Here, we identify and consider six mechanisms by which predators can increase parasitism in their prey populations (Figure 1), describing them in order from the mechanism most directly related to consumption of prey to the most indirect effects of predators. We also provide a set of theory‐ and evidence‐based heuristics with which to predict what mechanism may be at play—and, therefore, whether a particular predator is likely to increase parasitism in its prey. These heuristics are based on an understanding of the ways that prey, parasite, and predator traits, as well as aspects of the environment, predispose the system to include certain mechanisms. (Note: throughout this article, for simplicity, we use “prey” to indicate the prey/host species.) Finally, we provide guidance on how researchers can best select systems, design studies to investigate specific mechanisms, and report findings so that we can better understand and predict the outcomes of predator–prey–parasite interactions.

## 2 SLOPPY PREDATORS

Perhaps the most iconic predator–spreader in the ecological literature is the “sloppy predator.” The sloppy predator contributes to increased parasitism by consuming its prey in a way that increases the transmission or dissemination of parasites as compared to other sources of prey mortality (Cáceres et al., 2009; Duffy et al., 2019). The classic example of this phenomenon is the larval *Chaoborus* predator which shreds and regurgitates infected *Daphnia dentifera* prey (Cáceres et al., 2009; Strauss et al., 2016). This predation behavior spreads fungal parasite spores in the water column where they are ingested by foraging *D. dentifera*. In contrast, *D. dentifera* that die from parasite virulence rapidly settle out of the water column, limiting onward transmission of fungal spores. In this system, both prey biology and environmental stratification contribute to limit transmission after prey death from virulent effects of the parasite, while prey and predator behavior increase transmission after predation by the sloppy predator.

In general, predator behaviors that spread prey blood, tissue, or viscera are likely to contribute to transmission as long as prey behaviors expose them to these new sources of infection. These prey behaviors include indiscriminate grazing or filter feeding, as we see in the *D. dentifera* example, but also scavenging behaviors which may lead prey to consume conspecifics post‐predation when they would not otherwise. Because our focus in this review is on scenarios where the predator does not become infected, we are not considering cases of cannibalism. Similarly, prey biology and the interaction between environment and prey space‐use impact whether prey are more likely to encounter the parasite after predation or after death from virulent mortality. If prey shed few parasites during their life and/or if parasite shedding from live prey is concentrated in areas avoided by healthy prey, then there is a clear opportunity for sloppy predation to increase transmission. In fact, questions of spatio‐temporal patterns of predator spreading are likely of broad relevance to this topic (Box 1).

BOX 1 **Space and time.**

Predator–prey and parasite–host interactions both require temporal and spatial overlap between the victim and the natural enemy. Hosts must encounter other infected hosts or parasite transmission stages to allow transmission, while predators must encounter their prey to kill and eat them. Predator–prey–parasite interactions can increase parasitism in prey by increasing the spatio‐temporal overlap between prey and their parasites. In heterogeneous landscapes where parasites are deposited unequally across space, predator interference can increase the extent to which parasite deposition overlaps with areas of prey space‐use through both sloppy predation and parasites passing through predators (Cáceres et al., 2009; Duffy et al., 2019; Lopez & Duffy, 2021; Strauss et al., 2016); facilitation of prey–parasite spatio‐temporal overlap by the predator generally requires strong spatio‐temporal overlap between that predator and infected prey.

However, the presence of predators can influence parasitism in prey even without regular spatio‐temporal overlap between prey and predators (Clinchy et al., 2013). If prey and predators occupy the same areas at different times, then predator cues can have profound behavioral and physiological effects on parasitism in the prey even without any direct interactions. However, the extent of these effects is typically limited by the spatial scale and temporal duration of predator cues and the duration of parasite persistence in the environment. Highly localized predator cues (such as feces in terrestrial systems) may require far finer scale spatial overlap between prey and predator than more diffusible cues (such as predator kairomones in an aquatic system). Likewise, durable predator signals (such as a strong chemical cue) require less temporal overlap between prey and predator than more ephemeral signals (such as auditory cues). These behavioral effects of predators, themselves, may alter the space‐use of prey in ways that result in increased spatiotemporal overlap with parasites or infected prey. Similarly, parasites which persist longer in the environment are more likely to be encountered by prey than those with limited environmental viability, making predator spreader effects persist longer for these parasites.

The flexibility in the extent of spatiotemporal overlap required for predator–prey–parasite interactions—as well as the way in which the interaction itself alters spatiotemporal patterns of both prey and parasites—may make detecting patterns and inferring causal mechanisms challenging in some systems. Therefore, we recommend careful study of the locations and time periods during which contact between prey and parasites occur both to improve predictions about the potential effect of predators on those spatio‐temporal overlaps and to aid in measuring mechanistic intermediary factors between predator presence and parasite outcomes.

Efforts to discover and experiment on a wider array of sloppy predator–spreader systems should begin by identifying predatory behaviors that spread blood, tissue, or viscera. In these systems, field manipulation of predator presence/absence should be supported and contextualized with lab or field mesocosm simulation of predator sloppy eating byproducts to determine whether sloppiness mechanistically results in transmission.

## 3 PARTIAL PREDATION

In the simplest case of predator spreading via partial predation, the predator causes a wound that provides a direct route of entry for parasites. Moreover, devoting energy to repairing the wound renders prey less able to invest in immune function and other defenses against parasitism. In addition to this simple scenario, it can also be the case that the predator acts as a mechanical vector for disease. In these cases, the predator contaminates itself with the parasite (smeared on/in its mouth or other body parts) when it preys on an infected prey individual, and then exposes another individual by preying on but not killing it (practicing partial predation). In some ways, this latter scenario is similar to sloppy predation, but, in this case, the predator is directly bringing the parasite into contact with the prey. For the purposes of this review, we draw a distinction between this mechanical process of “vectoring” parasites between partially predated hosts and the more widely studied biological vectors such as mosquitoes which themselves become infected with the parasite (which is beyond the scope of this review). An iconic example of partial predation, and of partial predation predator spreading, is corals and their corallivorous predators. Corallivores are generally associated with increased disease in corals across a wide array of systems and have been experimentally demonstrated to “vector” infection from an infected coral to an uninfected one through partial predation (Renzi et al., 2022). Although these predators are sometimes considered alternate hosts or reservoirs of the infection (Aeby, 1998; Gignoux‐Wolfsohn et al., 2012), the actual mechanism for the parasite transfer is often uncharacterized (Clemens & Brandt, 2015) and the line between mechanically and biologically vectoring infection through partial‐predation is far from crisp. The importance of partial predation resulting in predator spreading in these systems stems from the sessile life‐style of the prey. Partial predators can spread infection over much longer distances than direct contact between prey and with much more specificity than passive dispersal through the water (Renzi et al., 2022). Partial‐predation predator spreading is not, however, restricted to sessile prey. For example, sub‐lethal parasitoids are known to frequently vector viral pathogens between prey individuals when ovipositing. However, even in this case, there is still a large difference in dispersal distance, given that parasitoids travel much more than larval prey (Cossentine, 2009). Plants are also sessile organisms which are frequently victims of partial predation; while they are generally beyond the scope of this review we discuss them briefly in Box 2.

BOX 2 **Herbivore spreaders?**

Although our framework focuses primarily on predation of animals, it is worth noting that herbivores can spread disease between plant victims by similar mechanisms. Herbivory exhibits much resemblance to predation with the notable difference that it rarely results in plant death. Due to this trait, partial predation herbivore‐spreading is better studied in plant‐herbivore systems than in predator–prey systems. For example, plant parasites frequently pass through herbivores such as beetles and are deposited back on the same or a different plant in feces (Wielkopolan et al., 2021). Saprozoic nematodes frequently ingest pathogenic bacteria from dead plant tissue and spread bacteria to new plant hosts by defecating in soil (Chantanao & Jensen, 1969; Nykyri et al., 2014). Although herbivores rarely kill whole plants, selective herbivory on uninfected individuals (Mauck et al., 2015), or on the basis of traits that correlate with parasite load such as size/growth rate (Cornelissen et al., 2008; Dietrich et al., 2005; Hoffland et al., 1996) are likely to decrease survival of uninfected plants, increasing disease in the population.

Although we rarely consider plants as having behaviors (but see (Karban, 2015)), they do display plastic responses to herbivory which have been demonstrated to increase susceptibility to parasites. For example, herbivore wounding frequently increases jasmonic acid production in plants which in turn can downregulate the production of salicylic acid, a common compound in defense against parasites (Bostock, 2005; Smith et al., 2009; Stout et al., 2006; Vlot et al., 2009). Many of these herbivore‐spreading systems, such as the partial predation of aphids or the physiological jasmonic acid/salicylic acid response are undoubtedly better characterized than any animal predator‐spreading systems with the accompanying complications and nuances. Therefore, these systems may provide useful parallels for future predator–spreader study. To that end, we suggest that researchers studying predator spreading and herbivore–parasite interactions acquaint themselves with the other body of literature.

The study of partial predation predator spreading is rich and continuing to develop rapidly. We suggest an additional, new focus on the importance of incidental partial predation or failed predation in predator spreading. Systems in which prey typically survive potentially lethal predation attempts may be prone to the transfer of parasites via contaminated mouthparts, talons, and claws. Regular wounding through partial predation may also produce a subset of the prey population whose immune defense systems are substantially compromised. As in the case of coral partial predation predator spreading, these processes should prove most important in systems with low contact or opportunities for direct transmission between prey.

**FIGURE 2:**
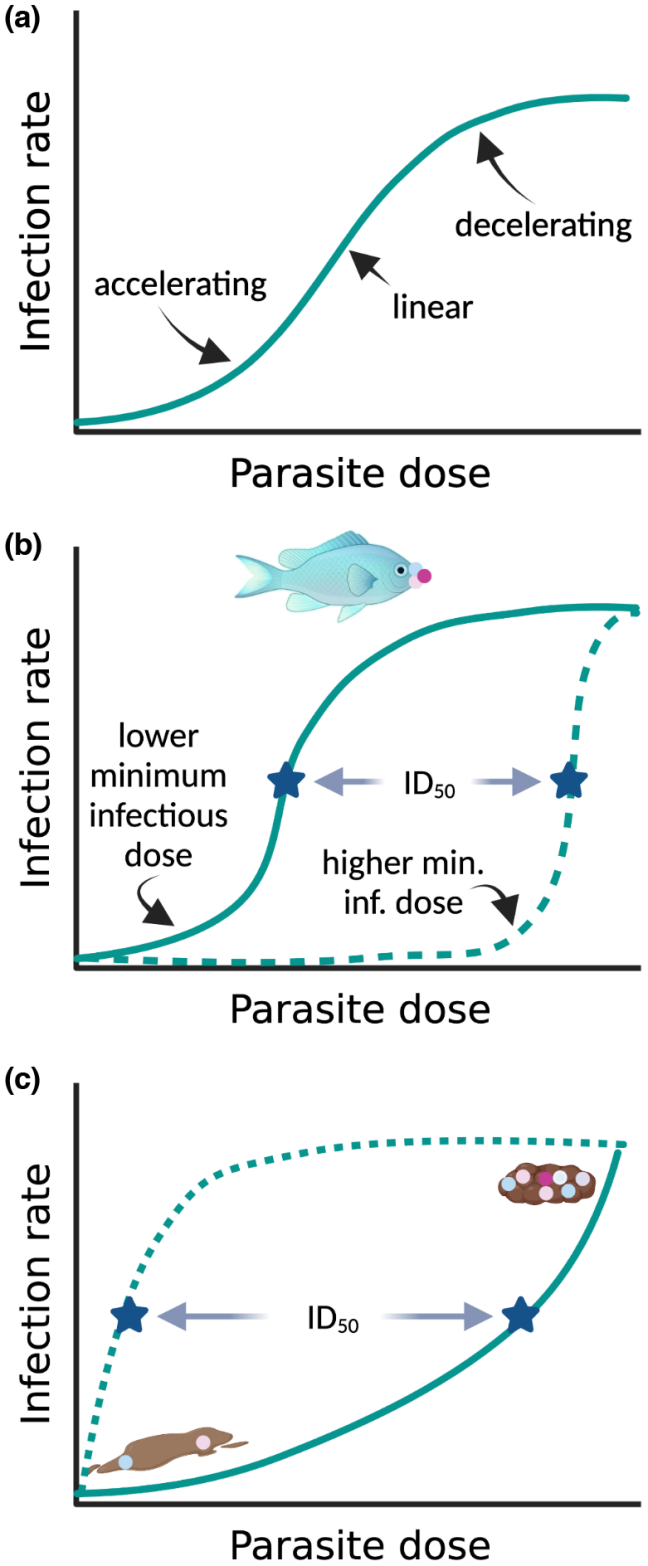
Dose‐infectivity relationships (a) can interact with partial predation (b) and whether parasites remain viable after passing through predators (c) to influence predator spreading. (a) Infection rate generally increases with increasing parasite dose, with a relationship that can be accelerating, linear, or decelerating. Moreover, as the curve shows, a sigmoidal dose–infectivity relationship can appear to be accelerating, linear, or decelerating depending on the particular range of parasite doses that are considered. (b) In the case of partial predation predator spreading, the impact of the predator on parasitism should depend on the nature of the dose–infectivity relationship. A partial predator (such as a corallivorous fish) that carries a moderate dose of the parasite between predators should be a very effective predator spreader for parasites with a low minimum infectious dose (and correspondingly low dose yielding 50% infections, known as the ID_50_ and indicated by a star on the figure), but not for those with a high minimum infectious dose (and ID_50_). (c) In cases where parasites pass through the predator's digestive tract, we expect there to be an interaction between the density of fecal material and the dose–infectivity curve: if there is a rapidly saturating dose–infectivity curve (and low ID_50_; dotted line) parasites passing through the digestive tract of a predator with diffuse feces (represented by the feces in the lower left of the figure) that spreads over a wide area should lead to substantial predator spreading. However, if there is an accelerating dose–infectivity curve and high ID_50_ (solid curve) predator spreading might be most pronounced when predators produce compact feces that contain a large number of parasites in comparison to the diffuse feces; in these cases, the predator spreading should be more localized. Created with BioRender.com.

Recent work has explored how the transport of parasites between contaminated locations by vectors (e.g., a pollinator in a plant–pollinator interaction) or by passive abiotic processes can influence levels of parasitism in the population, finding that the outcome depends on the dose–infectivity relationships (Ng et al., 2022) whether they be accelerating, linear, or decelerating (Figure 2a); the same should be true of partial predation predator spreading. In cases where the minimum infective dose is high, vector‐based spread does not result in as much parasitism as would be expected from a simpler compartment model (dashed line in Figure 2b) or as in cases with a low minimum infectious dose (solid line in Figure 2b). In contrast, passive abiotic processes, such as wind or water current dispersal, which contaminate additional sites can lead to faster than expected spread of disease when there is an accelerating dose–infectivity curve (that is, when the relationship between dose and infection is concave up, as shown in Figure 2a) (Ng et al., 2022); while accelerating dose–infectivity curves are not common, they do occur (Clay et al., 2021). These results suggest that it would be interesting to expand the work of Ng et al. (2022) to cases where predators are responsible for parasite spread and, as we discuss more in the next section (“Parasite passes through predator”), to consider the impact of dose–response relationships.

The study of partial predation predator spreading should focus on those systems with diffuse or patchy prey who frequently survive predation attempts. Direct testing of predator attack surfaces for parasites combined with tests of parasite persistence on similar surfaces should provide evidence for whether the predator is a viable spreader. Finally, experimental inoculation through wounding of prey could provide insights into this putative mechanism and nicely complement ecological studies of rates of infection after sublethal predation wounding.

## 4 PARASITE PASSES THROUGH PREDATOR

Predators, sloppy or not, tend to ingest many parasites along with prey tissues (Johnson et al., 2010). In some cases, these parasites are able to infect the predator and often this trophic transmission is obligatory for their life cycle (Kuris, 2003, 2005; Lafferty, 1999), making the predator also a host and beyond the scope of this review. However, many parasites are simply digested or, if they are more resilient, pass through the predator digestive tract and are excreted (e.g., fungal pathogens of *Daphnia* passing through fish guts or viral pathogens of spongy moths (*Lymantria dispar*) passing through avian guts; Duffy, 2009; Reilly & Hajek, 2012). Because many predators are larger and range more widely than their prey, this process of viable parasites passing through predators should promote the spreading of parasites to a larger number of prey over a wider spatial area and could even allow transmission between discrete prey populations. This mechanism is similar to partial predation in that the predator acts as a non‐host mechanical vector of the parasite, but in this case the predator only attacks the infected prey and spatially overlaps with the transmission target (Box 1).

Parasite resilience is the primary factor that facilitates this type of predator spreading as surviving the potentially harrowing passage through the predator gut is strictly necessary. Whether this condition is met is likely due to a combination of attributes of the parasite (e.g., a digestion‐resistant spore) and the predator (e.g., the pH of the digestive tract). If a parasite survives gut passage, predator and prey behavior then interact to determine whether this increases transmission. As noted above, if predators use more space than their prey, then parasites passing through predators should typically increase parasite transmission. However, several factors likely modulate this predator‐spreading effect. If prey preferentially avoid predator excrement (Apfelbach et al., 2005; Weinstein, Buck, et al., 2018), that should reduce transmission. In contrast, if prey seek out excrement for the nutrients it provides (Weinstein, Moura, et al., 2018), this behavior should promote predator spreading. Moreover, the form in which predators release feces (e.g., in a compact form such as a fecal pellet vs. more diffuse feces; Figure 2c) is likely to have an impact, though which type of feces leads to parasite spreading likely depends on multiple factors, including the nature of the habitat and prey behavior. For example, in a stratified lake where the prey are filter feeders (e.g., *Daphnia*), if predators release diffuse feces that contain infectious transmission stages, those transmission stages might be more likely to stay suspended in the water column where they can be taken up by a new prey individual; in this habitat type, releasing compact fecal pellets that sink out of the water column (as bluegill sunfish do) would likely reduce disease transmission (Duffy, 2009). In contrast, if other cases where the predator releases compact feces (e.g., as many terrestrial predators do), the nutrients in the more densely packed predator feces might increase primary production, attracting prey and increasing parasite transmission.

The dose–infectivity relationship is also likely to be important (Clay et al., 2021). In cases where the dose–infectivity curve is decelerating, the infectivity of each propagule decreases as they become more abundant. In these cases, the lower parasite densities that would be expected, on average, with diffuse feces might still be sufficient to infect many additional prey (as represented by the dashed line in Figure 2c). However, if the dose–infectivity curve is accelerating, adding more parasites leads to a greater increase in infections (as compared to a linear relationship where per spore infectivity does not change, and as shown by the solid line in Figure 2c); in these cases, more concentrated feces might lead to the highest infection levels, though this effect would likely be more geographically restricted. A recent meta‐analysis found evidence for accelerating, decelerating, and linear dose‐infectivity relationships, but found that decelerating relationships were most common (Clay et al., 2021)—which would mean that predators that release diffuse feces might, on average, be more likely to spread disease, since that feces would cover a wider area while still being infectious.

Study of predator spreading via parasites passing through predators requires the use of excrement analysis and necropsy of predators to identify parasites of their prey that they excrete but by which they are not infected. Ideally, these studies would seek not only to determine the presence/abundance of the parasite (e.g., via microscopy or molecular approaches), but also whether transmission stages are still viable. If a system has a parasite that survives gut passage and does not infect the predator, then a range of experimental manipulations could be devised to test whether the presence of a predator, or even just the predator's parasite‐laced feces, increases parasite transmission and/or facilitates transmission from one population to another. The role of a predator–spreader in such a system could also be detected using parasite genetic information: if parasites that pass through predators but have otherwise low mobility show minimal genetic structure over long distances with respect to prey movement then predator spreading may play a key role in transmission.

## 5 SELECTIVE PREDATION

Predators select their prey along a number of axes that can influence parasite loads and prevalences in the prey population. The healthy herds hypothesis was initially conceived on the basis that preferential predation on infected prey would lead to a decrease in parasitism at the population level (Hudson et al., 1992; Packer et al., 2003). It is reasonable to expect that infected individuals will often be more easily captured and eaten by predators (Hudson et al., 1992; Johnson et al., 2006). However, predators sometimes preferentially *avoid* consuming infected prey (Flick et al., 2016), for example, a study on black‐capped chickadees and downy woodpeckers found that they tended to avoid misshapen galls and unhealthy larvae, even during periods of food limitation (Schlichter, 1978). Indeed, a recent synthesis suggests that selective predation on uninfected individuals is more common than generally acknowledged (Gutierrez et al., 2022). In their original formation of the healthy herd hypothesis, Packer et al. (2003) noted that this type of selective predation should cause predators to increase parasitism in their prey.

**FIGURE 3:**
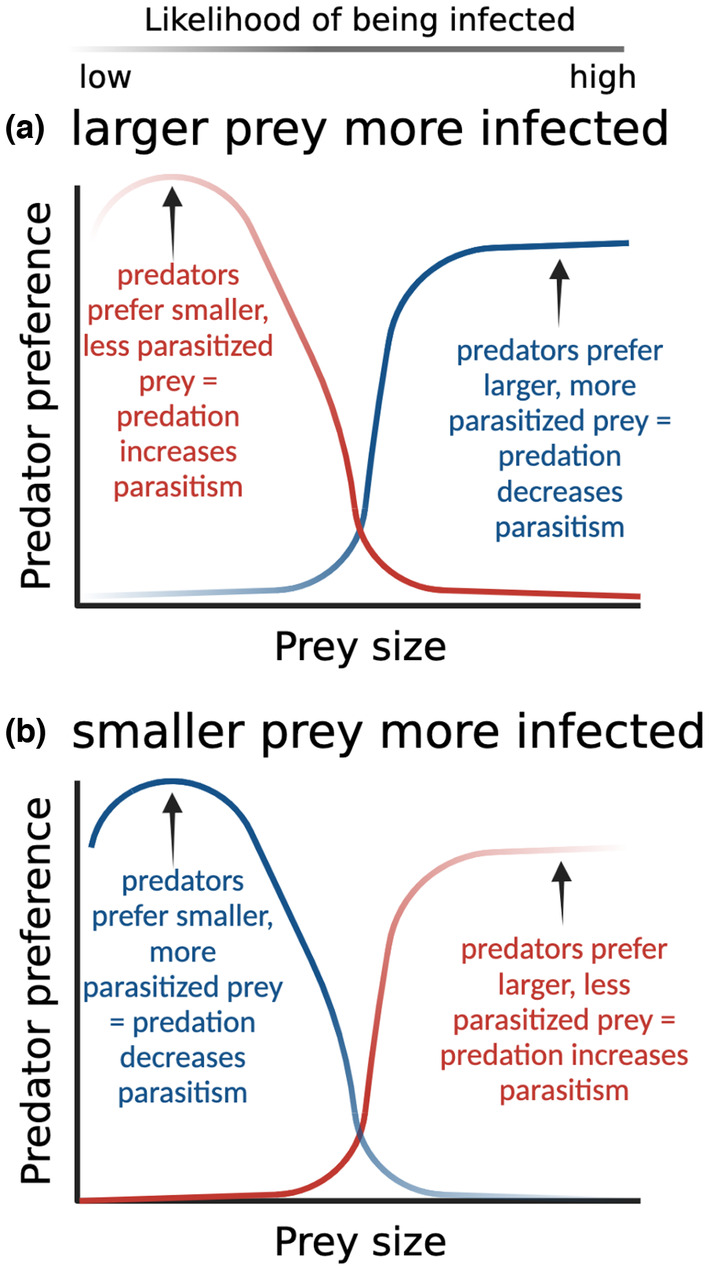
The impact of predator preference on infection levels depends on the relationship between the trait (in this example, prey size) and infection likelihood and between the trait and predation risk. If predators have a static preference for the type of prey that is more likely to be infected, that should reduce parasitism, as shown with the blue curves in a and b; if predators, instead, have a static preference for the type of prey that is less likely to be infected, that should drive predator spreading, as shown with the red curves. Created with BioRender.com.

Predators also select prey in a variety of other ways, leading to effects of consumption on disease that are more indirect but still potentially important. For example, many predators prey preferentially on particular sizes or ages of prey (King, 2002; Nilsson & Brönmark, 2000; Price, 1975), and prey sizes/ages commonly differ in their levels of infection (Dobson, 1989). These intersecting patterns can result in functional predator spreading via changes in prey population demography. For example, large intertidal snails (*Littorina littorea*) are far more likely to carry trematode infections than their smaller (and younger) conspecifics, but these larger snails are also far less likely to be preyed on by shell‐breaking predators such as crabs (Byers et al., 2015). As a result, predator preference for snail traits not directly related to infection resulted in much higher levels of parasitism in areas subjected to higher predation pressure (Byers et al., 2015), similar to the red curve in Figure 3a. A similar pattern has been found in hispid cotton rats (*Sigmodon hispidus*), which can grow too large for most avian predators. When large terrestrial predators are excluded, cotton rat size structure shifts toward larger rats, with no change in rat density; this shift is followed by an increase in the abundance of gastrointestinal nematodes that primarily infect larger rats (Richards et al., 2023).

This type of selective predation predator spreading requires a clear predation preference (e.g., due to innate or learned behavioral preferences and/or biomechanics) that is negatively correlated with the infection rates in prey (as in the red curves in Figure 3). Size/age may be the most straightforward version of this pattern, as in the case of the crab and rodent systems described above, as well as in systems where prey are attacked by a gape‐limited predator and a parasite that is more likely to infect larger individuals (as in the case of the *Chaoborus*‐*Daphnia*‐fungal parasite system (Cáceres et al., 2009)). However, many other possibilities for traits where there might be a negative correlation between infection rates and predation risk exist, such as sex (Acharya, 1995; Gwynne, 1987; Harrison et al., 2010; Krasnov et al., 2005; Lodé et al., 2004; McCurdy et al., 1998; Reimchen & Nosil, 2001), reproductive status (Tait et al., 2008; VanderWaal & Ezenwa, 2016), prey food choice (Garvey et al., 2020a, 2020b; Geervliet et al., 1996; Kester & Barbosa, 1994), species assemblage (de Rijk et al., 2013), and personality (Kortet et al., 2010). In any system where a trait increases the risk of predation but decreases the risk of parasitism, we expect predator spreading; conversely if there is a positive relationship between the trait and both infection risk and predation risk (as in the blue curves in Figure 3), we expect predation to have a healthy herds effect.

Much of this logic assumes that the prey classes that are selectively removed by predators simply disappear without any other effects on prey population dynamics. However, the removal of large classes of a population can have immense effects on the growth, development, and reproduction rates of other groups which can, in turn, influence infection. For example, the loss of large‐bodied prey may make resources available for smaller or younger individuals, increasing their growth, maturation, and reproduction rates (Abrams & Rowe, 1996; Relyea, 2007), potentially influencing population level parasitism rates in unexpected ways, especially since susceptibility to infection commonly changes with age, as reviewed in Ben‐Ami (2019). Predator behavior is also both plastic and adaptive which can result in preferences for prey classes that change according to prey availability or environmental cues (Coblentz, 2020; Johnson et al., 2006; Williamson, 1980).

As selective predators and unevenly distributed parasites are both ubiquitous, much additional experimental work is required to understand the interaction between these two processes. We encourage both predator–prey ecologists and disease ecologists to look at their study system from the alternative perspective and identify any potentially interacting patterns of parasite and predator selectivity. There are also likely extensive datasets on selective predation by human hunters with which these questions could be addressed (e.g., Chronic Wasting Disease in white‐tailed deer (Rivera et al., 2019)). This area would also benefit greatly from the combined usage of dynamical population modeling to make predictions for the outcomes of selective predation in a system and manipulative experiments to test these predictions (Box 3).

BOX 3 **Using mechanistic models to measure the impact of predators on parasite transmission.**

Individuals have been advocating for a relatively long time that ecologists and evolutionary biologists need to confront mechanistic models of ecological and evolutionary processes with data (e.g., Hilborn & Mangel, 1997; Turchin, 2003). Over the last 25 years, we as a field have taken that advice to heart particularly with regard to the study of disease ecology and evolution (e.g., Duffy et al., 2011; Dwyer et al., 1997; Elderd, 2019). This advice also holds true for thinking about how predation affects disease spread and incidence. While there are multiple mechanisms that we outline throughout the paper and potential pathways for predators to spread parasites, whether or not these mechanisms are important for determining the rate and the extent of parasite transmission will often come down to a series of statistical tests using standard statistical (e.g., linear regression) or mechanistic models. Here we advocate for a more mechanistic approach. As a heuristic, consider the following model of disease transmission where the prey becomes infected with a lethal parasite (Elderd & Dwyer, 2020),

(1) $$\frac{dS}{dt}=-\mathrm{\nu SP},$$

(2) $$\frac{dE}{dt}=\mathrm{\nu SP}-\mathrm{\gamma E},$$

(3) $$\frac{dP}{dt}=\mathrm{\gamma E}.$$

Here, *S* represents susceptible individuals, *E* represents exposed individuals, and *P* represents the parasites in the system. ν is the transmission rate and γ is the rate at which infected individuals are converted into parasites. In a field experiment, we can control the number of susceptibles and the number of parasites. If we conduct an experiment with a known number of parasites, we know *P* at time 0 or *P*(0). If the same experiment is conducted over a period of time from time 0 to time *T*, we can integrate Equation 1, so that:

(4) $$-\ln\left(\frac{S\left(T\right)}{S\left(0\right)}\right)=\mathrm{\nu P}\left(0\right)T.$$

Note that *S*(*T*)/*S*(0) can also be rewritten as 1 – *i* where *i* is the percentage of individuals infected.

Now, consider that we have added a predator to our experiment. The predator could either increase or decrease the spread of the disease. We could then add a term to Equation 4 that changes the transmission rate based on predator addition. The modified equation would read:

(5) $$-\ln\left(\frac{S\left(T\right)}{S\left(0\right)}\right)=\nu \;e^{\mathrm{\mu D}}P\left(0\right)T.$$

**FIGURE B1:**
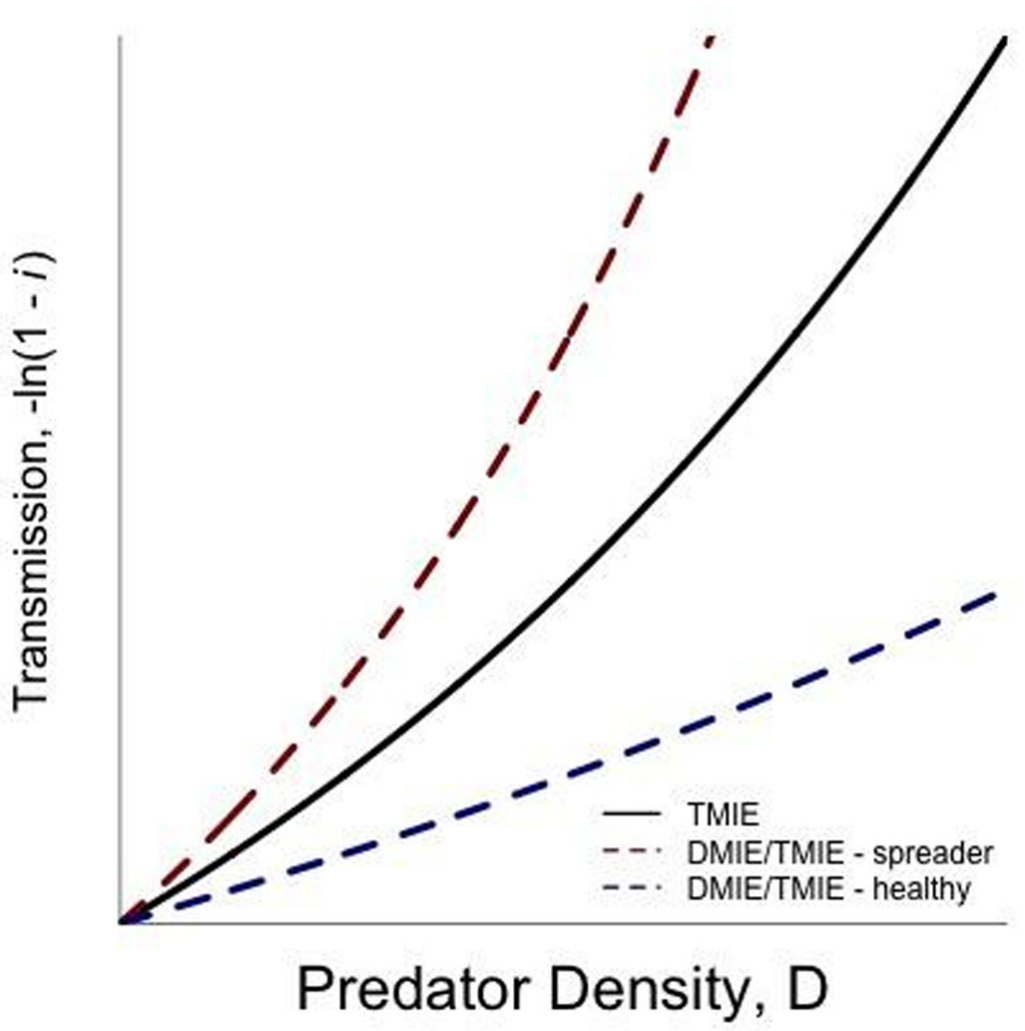
Effect of predator density (*D*) on parasite transmission (−ln(1 − *i*)). The solid line denotes an experiment where predators are added to the system but cannot directly change the amount of pathogen in the system and estimates of μ are driven by trait‐mediated indirect effects, $\mu_{\text{TMIE}}$. Here these TMIEs increase transmission as predator density increases. The dashed lines denote experiments where predators can alter the behavior or infection probability as well as change the amount of pathogen in the system, $\mu_{\text{DMIE}/\text{TMIE}}$. The upper dashed red line shows an increase due to the predators spreading the disease and the bottom dashed blue line shows a decrease via a healthy herds mechanism. Note that here the amount of pathogen across predator density treatments is constant. If we manipulated both pathogen and predator density, we would instead be estimating parameters associated with a surface.

Here the sign and the magnitude of μ dictate the effect of the predator on the system as a linear function of predator density, *D*. If μ = 0, the predator has no effect. If μ < 0, the predator essentially follows the healthy herd hypothesis by decreasing disease transmission. If μ > 0, the predator spreads the disease and increases disease transmission (Figure B1).

Here $\;e^{\mathrm{\mu D}}$ affects transmission either via changes in the transmission rate (e.g., changes contact rates between the prey and the parasite or probability of infection due stress) or changes in the amount of pathogen in the system. The former could be considered trait‐mediated effects and the latter could be considered density‐mediated effects. A simple series of experiments could then be designed to examine both trait‐mediated and density‐mediated indirect effects of predation on disease transmission. To test for trait‐mediated effects, past work has either exposed potential prey to predator cues (Buss & Hua, 2018; Yin et al., 2011) or modified the predator so that they are unable to depredate the prey (Schmitz et al., 1997; Wineland et al., 2015). This is the solid line in Box Figure, which shows that the presence of a predator increases disease transmission (i.e., a predator–spreader). A separate experiment could then expose the prey directly to predators who are able to directly alter the amount of pathogen in the system either by increasing the amount of pathogen (i.e., predator–spreader) or decreasing the amount of pathogen (i.e., healthy herds). These are the corresponding dashed lines above and below the solid line in Box Figure, respectively. Using the parameter estimates of μ for both the experiments, we can estimate the strength of the density‐mediated effect and the trait‐mediate effect through simple subtraction. For instance, we can estimate μ corresponding to the trait‐mediated only treatment ($\mu_{\text{TMIE}}$) and μ from the treatments where the prey are exposed directly to predators that account for both the trait‐mediated and the density‐mediated indirect effects ($\mu_{\text{DMIE}/\text{TMIE}}$). To estimate $\mu_{\text{DMIE}}$ or the effect of density‐mediated effects only, we simply subtract $\mu_{\text{DMIE}/\text{TMIE}}$ from $\mu_{\text{TMIE}}$. For the experiments considered above, because it is only possible to estimate transmission or infection rates using the animals that are recaptured, *S*(0) in the analysis corresponds to the number of susceptible individuals that are recaptured at the end of the experiment and *S*(*T*) corresponds to the number of individuals who have not become infected.

Thus, this simple addition to a standard mass‐action model of disease transmission can serve as a first pass on whether or not a predator will have an impact on transmission. Note that the model can be easily modified to consider specific aspects of the predator. That is, while here μ is a linear function of simply predator density, it can take on a variety of forms such as various non‐linear models whose terms differ based on experimental treatments or models based on a predator's functional response.

To confront the model with data, we can take a number of approaches; here we advocate two. The first approach is using standard information theory (Burnham & Anderson, 2002; Gotelli & Ellison, 2004) such as the Akaike Information Criterion (AIC), whereby we have multiple models that we directly compare. Since our solved model (Equation 5) only has two parameters to estimate, we can directly compare a model that just estimates the transmission rate ν to a model that estimates both the transmission rate ν and the effect of the predator via μ on transmission. The same approach can also be analyzed using a Bayesian framework and the appropriate model comparison metrics as the Watanabe Akaike Information Criterion (WAIC) (Hobbs & Hooten, 2015); this is the second approach that we advocate. The advantage of the Bayesian framework is not only the ability to compare multiple models but also the ability to derive a probability distribution associated with each of the transmission parameters estimated (Elderd, 2018).

## 6 CHANGES IN PREY BEHAVIOR

Prey organisms respond behaviorally to predation pressure in a variety of ways, many of which can increase parasite transmission, leading to behaviorally mediated predator spreading. These predator‐spreading behavioral changes are a type of “non‐consumptive” or “trait‐mediated” effect of predators on parasites via their prey (Daversa et al., 2021; Preisser et al., 2007; Schmitz et al., 1997, 2000). Behaviorally mediated effects tend to fall into one of two categories: (i) increased contact rates between prey individuals and (ii) decreased parasite avoidance behaviors. Behavioral predator spreading is relatively well studied in aquatic systems. Trinidadian guppies shoal in larger groups when under high predation and this higher group size increases transmission rates of *Gyrodactylus* parasites, resulting in higher burdens of this ectoparasite (Stephenson et al., 2015). Alternatively, wood frog tadpoles (*Lithobates sylvaticus*) increase their active time in the presence of trematode parasites to avoid infection; however, in the presence of parasites and predators, they decrease activity to avoid predation, increasing their susceptibility to trematode infection (Szuroczki & Richardson, 2012). Both these examples share a common pattern: a prey behavior that impacts parasite transmission is disrupted or altered by the presence of predators.

Behaviorally mediated predator spreading should be possible in many systems where prey alter behavior substantially in response to predators, and a few types of identifiable behavior modifications are most suspect. Any prey that displays conflicting parasite avoidance and predator avoidance behavior is likely to sacrifice one for the other when confronted with both natural enemies (Weinstein, Buck, & Young, 2018). Alternatively, predator response behavior that increases prey group sizes or interaction frequencies should directly increase parasite transmission through increased contact rates (Anderson & May, 1979). However, system‐specific knowledge about parasite–prey pairs may illuminate additional types of predator‐induced behavior that could amplify parasitism. Parasite‐induced behavioral changes are frequently discussed when they increase predation in trophically transmitted parasites (Kuris, 2003, 2005; Lafferty, 1999). Although such behavioral changes are generally considered maladaptive in non‐trophically transmitted parasites, mechanical predator‐spreading processes discussed above may result in a selective pressure for parasite behavior manipulation even in non‐trophically transmitted parasites. Moreover, rapid evolution in response to a predator may also promote parasitism, particularly if there is a tradeoff between behavioral resistance to predation and parasitism (Buss & Hua, 2018).

Behaviorally mediated predator spreading has been reasonably well studied in aquatic systems but relatively unstudied in terrestrial systems. However, similar behavioral changes could result in increased parasitism in terrestrial systems. For example, the beetle *Leptinotarsa decemlineata* is attacked by predators above ground but parasites belowground (Ramirez & Snyder, 2009); if beetles shift habitat use in response to predator cues, this behavior should increase parasitism. In another terrestrial example, an aphid (*Microlophium carnosum*) experiences increased parasitism by a fungal pathogen (*Pandora neoaphidis*) at the population level in the presence of coccinellid predators (Baverstock et al., 2009). When aphids perceive potential predators they flee by dropping from their host plant and subsequently colonizing the same or another plant. This process exposes the aphids to substantially more leaf surface area and therefore fungal spores than if they remained on a single leaf (Baverstock et al., 2008).

Researchers could specifically target systems where predator presence substantially alters prey space‐use behavior or contact networks to test the downstream effects on parasitism. Regardless of the system, it is extremely important to appropriately measure behavioral changes due to predator presence in a way that relates directly to how those behaviors may affect transmission.

## 7 CHANGES IN PREY PHYSIOLOGY/IMMUNITY

Prey responses to predator presence extend beyond the behavioral to the physiological, and these physiological changes can produce additional non‐consumptive effects of predators on parasites. Prey often decreases foraging behavior in favor of hiding or vigilance in response to predators (Brown et al., 1988; Creel et al., 2014; Jones & Dornhaus, 2011; Thaler et al., 2012). Predator presence also frequently increases hormonal stress levels in prey organisms (Cinel et al., 2020; Clinchy et al., 2011, 2013; Middlemis Maher et al., 2013). Both decreased nutrition and increased stress have been shown to have negative effects on organismal immune function which can in turn increase susceptibility to parasites (Hamilton, 1974; Martin, 2009; Navarro et al., 2004; Strandin et al., 2018; Viney & Riley, 2014). This results in a functional tradeoff between predator and parasite response in prey organisms (Adamo et al., 2017; Navarro et al., 2004; Otti et al., 2012). For example, house sparrows exposed to barn‐owl predators had a reduced T‐cell‐mediated immune responses and higher prevalence and intensity of *Haemoproteus* malarial infection in separate experiments (Navarro et al., 2004). However, complex immune system interactions are not required for physiology‐mediated predator spreading. In another example, predator kairomones induce *Daphnia dentifera* to grow larger and mature faster which in turn makes them more susceptible to infection by their yeast parasite (Duffy et al., 2011; Yin et al., 2011).

We predict that physiologically mediated predator spreading will be most evident where prey have large physiological or stress responses to predator presence leading to a diversion of resources away from immune function. Operationalizing this prediction requires an extensive understanding of prey physiology in order to identify systems in which predator stress downregulates immune function. However, there are a few heuristics that can be applied. In particular, predators with ambush or sit‐and‐wait predation strategies tend to be perceived as a larger threat/stressor than ranging predators, leading to a larger physiological response (Clinchy et al., 2011, 2013; Preisser et al., 2007). Additionally, prey that are highly energy‐ and/or nutrient‐limited even in the absence of predators are more likely to suffer physiological effects from the introduction of predators (Creel & Christianson, 2008).

Ideal systems for the study of physiologically mediated predator spreading are those in which physiological effects of predation are already well characterized and those in which predator presence can be manipulated without consumptive predation (e.g., by using a caged predator or a predator that has been rendered incapable of attacking prey, or by using predator chemical cues; e.g., Buss & Hua, 2018; Duffy et al., 2011; Flick et al., 2020; Schmitz et al., 1997; Szuroczki & Richardson, 2012). Experiments in particular require measuring not just the predator effect on a parasite response but also the effect of predator exposure on at least one physiological intermediary. Easier to measure non‐invasive intermediary metrics include body condition and fecal stress hormones (Palme et al., 2005; Sánchez et al., 2018) but ideally a study would measure both this type of proximate response to predators and the ultimate effect of predation stress on immune function. Such experiments are likely to be expensive and time intensive but hold the key to a potentially underestimated type of predator spreading.

## 8 BEYOND SINGLE MECHANISM STUDIES

Each of the individual mechanisms for predator spreading described above requires substantial additional study to understand both their relative importance across natural systems and the factors that contribute to the strength of the effect (Box 4). While much can be learned by studying a single mechanism in a single predator–prey–parasite system, a broad understanding of the importance of predator spreading to disease dynamics in wildlife requires inference that spans mechanisms and study systems (Duffy et al., 2021). This type of inference necessitates both the study of multiple predator‐spreading mechanisms within a single system and the comparison of predator spreading across a wide range of biological systems.

BOX 4 **Predictions and Outstanding Questions**

*Sloppy predators*

We predict predator spreading if:

- transmission after death from parasitism is limited
- prey and/or predator behavior lead to increased transmission after predation

Outstanding questions:

- Is sloppy‐predator spreading more common in aquatic systems?
- Are certain parasite taxa, transition modes, or infection sites more prone to sloppy‐predator spreading than others?
- Are scavengers and cannibals more or less likely than grazers to experience sloppy predator spreading?

*Partial predation*

We predict predator spreading if:

- predator contaminates itself while feeding on one prey item
- predator wounds, but does not completely consume, prey
- Wounding of the prey decreases general defenses against disease or parasite spreads through open wounds

Outstanding questions:

- Is partial predation predator spreading limited to sessile/colonial organisms such as coral?
- What parasite taxa, transmission modes, or infection sites are most prone to this form of transmission?
- Are there systems in which failed predation events are infectious and common enough to influence disease dynamics?

*Parasite passes through predator*

We predict predator spreading if:

- (at least some) parasites can survive gut passage
- predator range is substantially larger than prey range
- prey preferentially feed in areas where predators defecate or predator feces are widely distributed

Outstanding questions:

- What taxa of parasites are most likely to survive predator gut passage?
- What taxa of predators or predation strategies are most likely to produce increases in transmission due to parasites passing through predators?
- What aspects of predator physiology are likely to promote parasites surviving gut passage and/or effective spreading of infectious stages of a parasite?

*Selective predation*

We predict predator spreading if:

- Predators selectively consume prey that are less likely to be infected (either actively avoiding infected prey or due to selection on traits that correlate with parasitism, such as body size)

Outstanding questions:

- What types of predator selection preferences result in predator spreading?
- What taxa and aggregation patterns of parasites result in selective predation predator spreading?

*Changes in prey behavior*

We predict predator spreading if:

- A behavior that limits parasite transmission is disrupted or altered by the presence of predators
- The presence of predators increases contact rates or group sizes of prey

Outstanding questions:

- What predator avoidance behaviors are most prone to increasing parasite transmission?
- What prey taxa are most susceptible to this type of predator spreading?
- Is behavior‐mediated predator spreading more likely for certain types of parasites (e.g., ectoparasites)?
- Can the ability to effectively balance predator and parasite risk with behavior be selected for, or do prey organisms run up against fundamental constraints?

*Changes in prey physiology/immunity*

We predict predator spreading if:

- Predators cause prey to become energy‐ or nutrient‐limited
- Predators increase prey stress, diverting resources from immune functions

Outstanding questions:

- Do particular predation strategies lead to larger physiology‐mediated predator spreading?
- Are physiology‐mediated predator‐spreading effects of the same scale as other predator‐spreading effects?
- Does the strength of physiology‐mediated predator spreading vary with prey immune strategies?

Although we have noted the characteristics of systems that may make them especially suited to studying a particular type of predator spreading, it is vital that a variety of predator‐spreading mechanisms are tested in a single system. It is natural for a particular system to be used repeatedly to investigate a particular mechanism of predator spreading due to demonstrated feasibility. For example, behavioral predator spreading has been well studied in aquatic frog tadpole prey because tadpoles can readily be exposed to caged predators or predator kairomones to provoke behavioral responses, and because methods are well‐established (Buss & Hua, 2018; Han et al., 2011; Koprivnikar & Urichuk, 2017; Szuroczki & Richardson, 2012). It is, however, plausible that other predator‐spreading mechanisms such as sloppy‐predation, selective predation, and parasites passing through predators could prove important in some tadpole–parasite systems. By comparing the relative magnitude of effect sizes across multiple predator‐spreader mechanisms or by manipulating multiple mechanisms within a single experiment (e.g., by manipulating predator kairomone presence and the presence of shredded infected tadpoles), we can come to understand whether predator spreading is important to disease dynamics; this would also allow us to understand which mechanisms are key to driving this phenomenon in a particular system and whether or not there are synergistic effects between mechanisms. Long‐term, in‐depth, study of a single system across a broad range of mechanisms should also open doors to a better understanding of the role of spatiotemporal overlap in predator spreading (Box 1).

In addition to studying multiple mechanisms in a single system, we must study the same mechanism in multiple systems—an approach known as “horizontal integration” (Travis, 2006). Horizontal integration requires a range of systems that are amenable to study (Duffy et al., 2021); even without considering predation, dominant model systems in the study of disease ecology and evolution have major gaps that likely impede our understanding (Wale & Duffy, 2021). Despite these challenges, a comprehensive understanding of the factors that promote predator spreading will require studies that use prey from a range of taxa, predators that differ in key traits (e.g., related to how prey are detected and captured), with a variety of parasites (including microparasites, macroparasites, and ectoparasites), and in a variety of habitats (including terrestrial and aquatic). The development of generalized mathematical models with which to generate and test predictions across systems and mechanisms will be vital in integrating these horizontal and vertical approaches to predator spreading (Box 3).

Comparative analyses of predator‐spreading mechanisms across taxa and study systems will require substantial scientific coordination. At present, quite a few labs across the world have conducted or regularly conduct experiments or observational studies of predator–prey–parasite interactions. Some of these labs have a primary focus on predator–prey or parasite–host ecology and use a study system as a way of asking fundamental ecological questions. However, many predator–prey–parasite experiments are conducted with an aim toward better pest management in agriculture or other applied outcomes (e.g., Chacón et al., 2008; Chailleux et al., 2017; de Lourdes Ramírez‐Ahuja et al., 2017). Unfortunately, few of these studies share common reporting standards and many suffer from similar experimental design limitations. In addition to placing all data in a publicly accessible repository, we encourage future researchers measuring predator spreader effects to follow these general guidelines:

1. Report all measurable prey/parasitism outcomes; ideally at least prevalence and prey population density, and intensity if feasible
2. Measure as many proximate predator effects as is practical (e.g., prey demography, prey physiology, prey immunity, prey space‐use or grouping behavior)
3. Test a range of predation pressures instead of the presence/absence of predators

Most studies of predator–parasite interactions report only a single parasite outcome and rarely report prey population density (though studies on agricultural pests are a notable exception to the latter (Agboton et al., 2013; Chailleux et al., 2017; Kaneko, 2006)). The choice of parasite outcome, typically prevalence or intensity, is at times motivated by underlying theory or parasite biology, but frequently the reasons for the choice are unclear. Measuring intensity is more common in studies of macroparasites, but we suggest that it could be interesting to measure this for microparasites as well (since, e.g., predators may shift age or size structure in a way that alters the average burden of infection). We also note that the term “intensity” can denote different types of parasite quantification in different systems (similar to the case for the term “virulence”), so it will be important to clearly define how intensity is being quantified when reporting the results. Mechanisms of predator spreading affect prevalence and intensity in different ways (Richards et al., 2022). For example, if predators have the largest physiological effect on prey already susceptible to infection then individuals who are likely to be infected may become more heavily infected at a higher rate than healthy individuals become newly infected. The combination of effects on prevalence and intensity may also help to identify the specifics of predator‐spreading mechanisms. We also suggest the reporting of prey population density as an outcome because of the important role it plays in the original healthy herds hypothesis and our general understanding of the predator–prey–parasite interaction, and because this metric can be of particular interest (e.g., when the prey is of conservation concern) (Duffy et al., 2019; Packer et al., 2003). If predators increase parasitism but fail to have persistent effects on prey population densities or prey fitness, the predator–spreader interaction would be of limited use for understanding prey population dynamics. Studies on agricultural arthropod pests which manipulate both predators and parasites typically focus on prey density as the key outcome of interest (Agboton et al., 2013; Kaneko, 2006; Lin et al., 2019; Vance‐Chalcraft et al., 2007); these studies often focus on whether predator and parasite effects on pest species are additive, substitutive, antagonistic, or synergistic in order to best accomplish biological control of pest species (Lin et al., 2019; Roy et al., 2001; Zhang et al., 2015). Measuring effects on population density does generally require longer‐term studies than those simply reporting parasite outcomes, but the amount of additional time required varies substantially with prey life history. The reporting of all three of these outcome variables (prevalence, intensity, and density) will both improve understanding of mechanisms in individual studies and facilitate future synthetic and meta‐analytic work on the subject.

In addition to measuring and reporting multiple parasitism outcome variables, we also encourage researchers to measure and report as many proximate predator effects as feasible. These proximate predator effects include many of the intermediary mechanistic steps we detail above: prey demography, prey immune function, and prey space‐use behavior. Very few predator–parasite studies report any proximate predator effects but those that do are able to tell the clearest and most convincing stories of predator‐spreading mechanisms (e.g., Cáceres et al., 2009; Navarro et al., 2004; Szuroczki & Richardson, 2012). If investigating a particular mechanism of predator spreading, we hold it is essential to measure the proximate predator effects that mediate that mechanism. Moreover, because multiple predator‐spreading mechanisms are likely at play in any given system, future research would greatly benefit from casting a wide net of measured proximate predator effects when logistically feasible.

Nearly all predator–parasite studies include just two levels of predation, typically presence/absence or high/low (Richards et al., 2022), but it is well known that reducing a continuous spectrum of a predictor variable to a binary is dangerous for inference (Inouye, 2001). What may appear to be a clear positive or negative effect when considered at just two levels may in fact be a complex non‐monotonic relationship. Predator–parasite studies that consider a broader range of predation pressure levels have, in fact, found hump‐shaped relationships between predation and parasitism (Hawlena et al., 2010), as has been predicted by theory (Holt & Roy, 2007). It is also likely that some predator‐spreading mechanisms may operate most strongly at different points on the predator‐pressure spectrum. For example, behavioral effects may respond strongly to the introduction of predators but weakly to increases in predation pressure thereafter, whereas consumptive effects may respond more linearly with increasing predation pressure. In such a situation, multiple interacting predator–spreader effects may produce unexpectedly non‐linear relationships between predation pressure and parasitism over the full spectrum of predation.

## 9 CONCLUSIONS

The potential for predators to protect their prey populations from the harmful effects of parasitism is intuitive and has promising conservation and public health implications (Ostfeld & Holt, 2004; Packer et al., 2003). In agricultural pest systems, management sometimes has the opposite goal: using predators to facilitate pathogen spread to better limit pest abundances (Lin et al., 2019; Roy et al., 2001; Zhang et al., 2015). However, there is surprisingly mixed evidence for the effect of predators on parasitism in their prey, with roughly as many studies finding that predators increase disease as finding a decrease (Richards et al., 2022). This variability raises the question of what factors lead to predators reducing disease versus spreading it—a question that we must be able to answer with confidence if we wish to manipulate predation as a management strategy. Attempts to manage infectious diseases in wildlife based on incomplete understanding of natural systems can have catastrophic outcomes, as when culling of badgers in the United Kingdom repeatedly led to an increase in bovine tuberculosis and not the hoped for decline (Donnelly et al., 2003, 2006, 2007). Unfortunately, at present, we are far from being able to confidently predict who will be a predator spreader. However, the mechanisms that we identify in this perspective provide a framework for rigorously studying predator spreading; we also provide initial hypotheses regarding factors that should promote predator spreading via these different mechanisms, and guidance for how to carry out studies on this topic in the future (Box 4). These studies—and consistent, comprehensive results reporting—are essential if we are to better understand this phenomenon that is of basic interest and applied importance. We often learn the most when predictions break down and unexpected outcomes occur. While we expect many of our predictions here will prove reasonably sound, we anticipate that the most exciting research questions in the next generation of predator–prey–parasite ecology will grow out of discovering the places where these predictions fall apart.

## AUTHOR CONTRIBUTIONS

**Robert L. Richards:** Conceptualization (equal); investigation (equal); visualization (equal); writing – original draft (lead); writing – review and editing (equal). **Bret D. Elderd:** Conceptualization (equal); investigation (equal); visualization (equal); writing – review and editing (equal). **Meghan A. Duffy:** Conceptualization (equal); investigation (equal); visualization (equal); writing – review and editing (equal).

## FUNDING INFORMATION

This work was supported by the US National Science Foundation (1316334 to BDE and 1655856 to MAD) as well as by USDA grant 2019‐67014‐29919 to BDE as part of the joint NSF–NIH–USDA Ecology and Evolution of Infectious Diseases program, and by the Gordon and Betty Moore Foundation (GBMF9202 to MAD; DOI: https://doi.org/10.37807/GBMF9202).

## Data Availability

This article contains no data.
